# Mitochondria-Enriched Extracellular Vesicles (EVs) for Cardiac Bioenergetics Restoration: A Scoping Review of Preclinical Mechanisms and Source-Specific Strategies

**DOI:** 10.3390/ijms262211052

**Published:** 2025-11-15

**Authors:** Dhienda C. Shahannaz, Tadahisa Sugiura, Taizo Yoshida

**Affiliations:** 1 Faculty of Medicine, Universitas Indonesia, Jakarta 10430, Indonesia; dhiendaladdynasrul@gmail.com; 2 Department of Cardiothoracic and Vascular Surgery, Montefiore Medical Center/Albert Einstein College of Medicine, Bronx, NY 10467, USA; 3 Department of General Surgery, Montefiore Medical Center/Albert Einstein College of Medicine, Bronx, NY 10467, USA

**Keywords:** cardiac regenerative therapy, extracellular vesicles (EVs), induced pluripotent stem cell (iPSCs), mitochondrial transfer, cardiomyocyte repair, heart failure, stem cell-derived exosomes, mitochondrial dysfunction, regenerative medicine, targeted organelle delivery

## Abstract

Mitochondrial dysfunction is a pivotal contributor to cardiac disease progression, making it a critical target in regenerative interventions. Extracellular vesicles (EVs) have recently emerged as powerful mediators of mitochondrial transfer and cardiomyocyte repair. This review highlights recent advancements in EV bioengineering and their applications in cardiac mitochondrial rescue, with a particular focus on EVs derived from induced pluripotent stem cell–derived cardiomyocytes (iPSC-CMs). Drawing upon a growing body of preclinical evidence, we examine the mechanisms of mitochondrial content delivery, EV uptake dynamics, and comparative bioenergetic restoration outcomes across EV sources. Special emphasis is placed on therapeutic outcomes such as adenosine triphosphate (ATP) restoration, reactive oxygen species (ROS) modulation, and improvements in contractility and infarct size. The convergence of mitochondrial biology, stem cell-derived EV platforms, and engineering innovations positions mitochondria-enriched EVs as a promising non-cellular regenerative modality for cardiovascular disease.

## 1. Introduction

Cardiovascular diseases (CVDs) remain the leading cause of mortality globally, accounting for approximately 17.9 million deaths annually, representing 33% of all global deaths [1,2]. In the United States, CVDs are responsible for about 697,000 deaths each year, constituting 20.1% of all deaths [3,4]. Japan, despite its high life expectancy, reports that heart diseases are the second leading cause of death, following cancer [5,6]. In Indonesia, CVDs account for 35% of all deaths, underscoring a significant public health challenge [7,8]. A pivotal factor in many cardiac pathologies is mitochondrial dysfunction, which impairs cardiomyocyte energy metabolism and contributes to heart failure [9]. Addressing this issue necessitates innovative therapeutic strategies to restore mitochondrial function and enhance cardiac repair mechanisms.

Mitochondrial dysfunction underlies cardiac disorders such as ischemic heart disease, heart failure, and cardiomyopathies, disrupting oxidative phosphorylation (OXPHOS) and ATP production [9], which cardiomyocytes rely on for over 95% of their energy needs [9]. Impaired mitochondria induce energy deficiency, excessive ROS generation, calcium dysregulation, and apoptotic cascades, exacerbating myocardial damage [9]. Current treatments like beta-blockers and Angiotensin-Converting Enzyme (ACE) inhibitors do not restore mitochondrial integrity [10,11,12,13], highlighting the need for novel approaches. EVs, lipid bilayer-enclosed vesicles, mediate intercellular communication by transferring bioactive cargo—including proteins, miRNAs, lipids, and functional mitochondria [14]—offering a potential strategy for mitochondrial repair and cardiomyocyte survival.

Recent studies have explored the potential of extracellular vesicles (EVs) derived from induced pluripotent stem cell-derived cardiomyocytes (iPSC-CMs) [15] to deliver functional mitochondria to injured myocardium. For instance, research demonstrated that mitochondria-rich EVs could transfer healthy mitochondria into recipient cardiomyocytes, restoring bioenergetics and improving cardiac function in ischemic models [16,17]. Mitochondria-rich EVs restore ATP production, enhance oxidative metabolism, and suppress apoptosis, improving cardiac function in preclinical models of myocardial infarction and heart failure.

An MSC-EV loaded with mitochondria study showed a 38% left ventricle ejection fraction (LVEF) increase in ischemia–reperfusion rodent models [18,19], while iPSC-CM-derived EVs enriched with miR-133 and miR-1 enhanced cardiomyocyte survival and proliferation in vitro [20,21]. However, mitochondrial transfer mechanisms, uptake efficiency, and long-term benefits remain unclear, hindering generalizability and limiting clinical translation [22].

To address these gaps, this scoping review was conducted with the following objectives: to chart and consolidate current evidence on extracellular vesicle (EV) engineering for targeted mitochondrial therapy in cardiomyocyte regeneration. This review explores consistent patterns, highlights discrepancies across studies, and outlines functional and mechanistic domains relevant to therapeutic potential. It maps how engineered EVs may support bioenergetic restoration in dysfunctional cardiomyocytes through mitochondrial delivery—contributing to ATP recovery, oxidative stress reduction, and improved contractility.

The central aim of this manuscript is to synthesize and contextualize preclinical findings on EV-mediated mitochondrial therapeutics, focusing on EV isolation techniques, mitochondrial cargo characterization, delivery dynamics, and cardiomyocyte-targeted efficacy. This includes mapping methodological strategies—such as EV bioengineering approaches, mitochondrial labeling systems, and functional readouts including ATP levels, ROS modulation, infarct size reduction, and LVEF improvement.

Special attention is given to mechanistic pathways such as EV uptake modes, bioenergetic rescue mechanisms, and the fidelity of mitochondrial transfer. Additionally, this work contrasts iPSC-CM and mesenchymal stem cell (MSC)-derived EVs to delineate source-specific advantages and challenges. Positioned within a regenerative cardiology framework, this review aims to bridge translational gaps, inform next-generation EV development, and support more standardized preclinical models for future cardiac repair applications.

## 2. Materials and Methods

This review was registered in the International Prospective Register of Systematic Reviews (PROSPERO) under registration number CRD42011089458. No protocol amendments were made after registration. An internal protocol guided study screening and data charting, which is available upon request.

**Figure 1:**
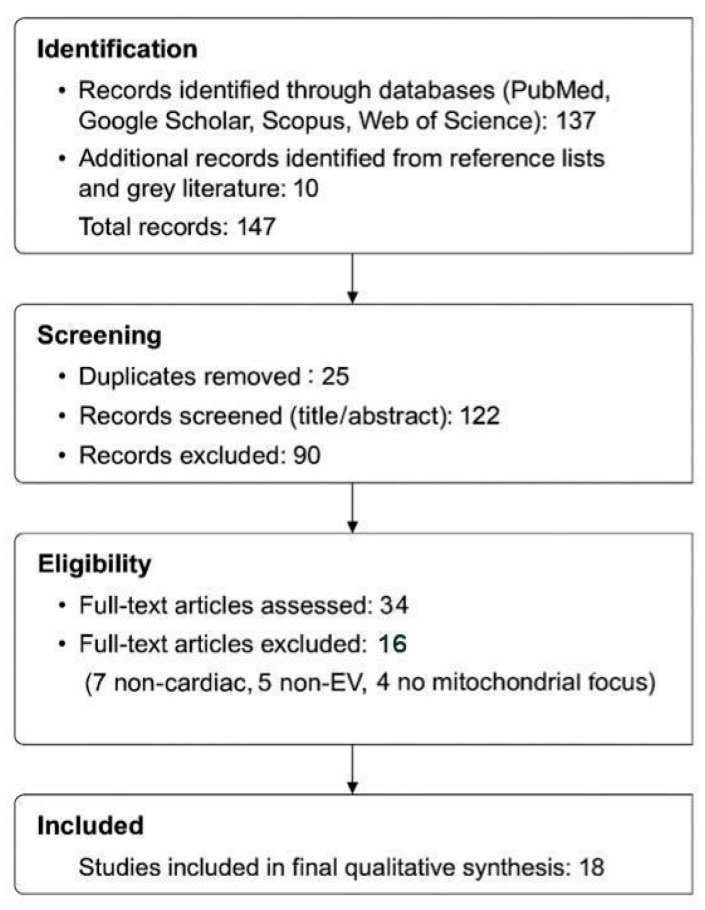
PRISMA-ScR Flow Diagram of Study Identification, Screening, and Inclusion Process.

To conduct this scoping review on extracellular vesicle (EV)-mediated mitochondrial transfer in cardiomyocyte regeneration, we followed the PRISMA-ScR 2018 guidelines (Figure 1). Literature searches were performed in PubMed, Google Scholar, Web of Science, and Scopus through June 2025. The following keyword combinations were used with Boolean operators:

(“extracellular vesicles” OR “exosomes” OR “microvesicles”) AND (“mitochondria” OR “mitochondrial transfer”) AND (“cardiomyocyte” OR “iPSC-CM” OR “MSC”) AND (“regeneration” OR “cardiac repair”) AND (“ATP” OR “ROS” OR “LVEF”).

Eligibility Criteria:

Studies were included if they met the following criteria:

(1) Preclinical in vitro or in vivo research investigating EV-mediated delivery of mitochondrial components to cardiomyocytes;
(2) Reported functional outcomes such as ATP production, ROS reduction, LVEF improvement, infarct size, or calcium handling;
(3) Described EV engineering or isolation methods (e.g., ultracentrifugation, surface modification, mitochondrial tagging);
(4) Utilized iPSC-CMs, MSC-derived vesicles, or primary cardiomyocytes as source or recipient cells;
(5) Were published in English between 2015 and 2025 to reflect contemporary bioengineering techniques.

Exclusion Criteria:

(1) Reviews, editorials, or commentaries without original experimental data;
(2) Studies lacking mitochondrial-specific analysis;
(3) Articles not focused on cardiac or cardiomyocyte models. However, select non-cardiac studies with clear mechanistic relevance to mitochondrial transfer were retained for contextual discussion under a separate section (“Emerging insights from non-cardiac models”).

### 2.1. Data Charting and Synthesis Approach

Data were independently charted by two reviewers (Tadahisa Sugiura and Dhienda C. Shahannaz) using a standardized charting form. Key variables extracted included:

(1) EV source (e.g., iPSC-CM, MSC, CPC)
(2) Engineering or modification method (e.g., passive loading, overexpression, electroporation).
(3) Mitochondrial cargo type (e.g., mtDNA, ATP5a1, PGC-1α).
(4) Recipient models and detection methods (e.g., flow cytometry, fluorescence microscopy).
(5) Functional endpoints (e.g., ATP restoration, ROS reduction, infarct size, LVEF).
(6) Safety and biodistribution metrics, if available.

Outcomes were extracted as reported, including fold change (ATP), percentage changes (infarct size, ROS), or directional improvements (LVEF). Where multiple time points or measurements were provided, the most translationally relevant data were prioritized. No statistical conversions or imputations were applied. Missing or unclear fields were recorded as “not specified” and only included if primary inclusion criteria were met.

Although formal grading tools (e.g., GRADE) were not applied due to the preclinical scope and heterogeneity of designs, consistency and methodological rigor across studies were qualitatively evaluated. Bias risk was assessed using a custom-modified SYRCLE™ tool (Redbound University Medical Center, Nijmegen, The Netherlands), with attention to randomization, blinding, quantification of EV dose, and endpoint validation. No automation tools were used, and no authors were contacted for data clarification.

### 2.2. Results Mapping and Synthesis Strategy

Due to the methodological diversity across included studies—ranging from EV isolation protocols and mitochondrial labeling to recipient models—a narrative synthesis was employed. Studies were categorized by:

- EV origin (iPSC-CM vs. MSC);
- Mitochondrial content (whole organelles vs. isolated proteins or mtDNA);
- Outcome domains (bioenergetic function, ROS reduction, LVEF, infarct size);
- Delivery mechanism (e.g., macropinocytosis, TNTs, clathrin-mediated endocytosis).

Where applicable, data were harmonized to percent or fold-change relative to controls for visual clarity. No meta-analyses or pooled estimates were performed.

Tables in Section 3 summarize comparative findings across included studies. Figure 1 (PRISMA flow diagram) outlines the full selection process. No sensitivity analyses or statistical heterogeneity tests were conducted, as this review does not aggregate results quantitatively. Internal consistency in the directionality of functional improvements (e.g., increased ATP or reduced infarct size) was considered qualitatively during synthesis.

### 2.3. Study Selection Summary

Out of 137 initially identified records, 122 remained after duplicate removal. Following title and abstract screening, 34 articles underwent full-text review. Of these, 18 met all inclusion criteria and were included in the final synthesis. Screening was performed independently by two reviewers, and discrepancies were resolved by discussion and consensus.

Sixteen studies were excluded during full-text review for the following reasons:

- Non-cardiac model (*n* = 7): e.g., EV-based mitochondrial therapies in glioblastoma, kidney injury, or NSCLC [23,24,25,26,27,28,29].
- Non-EV mitochondrial delivery system (*n* = 5): e.g., liposomal, nanoparticle, or MITO-Porter approaches [30,31,32,33,34,35].
- Lack of mitochondrial relevance despite EV or cardiac focus (*n* = 4): e.g., general EV studies without mitochondrial functional assays [36,37,38].

Reasons for exclusion are described, and excluded studies are cited accordingly for transparency.

## 3. Results

A total of 18 studies were included (Table 1), focusing on the role of extracellular vesicles (EVs) in delivering mitochondrial cargo or modulating mitochondrial function in cardiomyocytes and cardiac injury models. The primary EV sources were iPSC-derived cardiomyocytes (iPSC-CMs), mesenchymal stem cells (MSCs), cardiac progenitor cells (CPCs), and iPSC-derived endothelial cells (iPSC-ECs). The cargoes consisted of intact mitochondria, mitochondrial proteins such as ATP5a1 and TOM20, and regulatory miRNAs, including miR-144, miR-9-5p, and miR-202-5p (Studies 2, 4, 9, 7, 10, 18).

Mechanistic insights spanned multiple delivery and regulatory pathways, including endocytosis, AMPK/Akt-induced autophagy, and mitochondrial uptake via membrane fusion or gap junction mediation (Studies 8, 17, 18). Functional outcomes included enhanced ATP production and left ventricular ejection fraction (LVEF), as well as reductions in reactive oxygen species (ROS), apoptosis, pyroptosis, senescence, and infarct size (Studies 2, 6, 7, 10, 16).

### 3.1. EV Isolation and Engineering Approaches

EVs were predominantly isolated from conditioned media using differential ultracentrifugation, tangential flow filtration, or size exclusion chromatography (Studies 2, 10, 17). Density gradient methods yielded high-purity vesicles, whereas tangential flow systems allowed larger-scale processing. Surface engineering approaches included ligand conjugation and PEGylation to enhance cardiac targeting (Studies 16, 18). Mitochondrial enrichment was achieved through donor cell preconditioning, such as hypoxia or mitochondrial stimulation (Study 16). No studies in this review reported direct electroporation of mitochondria into EVs; instead, functional mitochondrial loading was achieved through endogenous packaging or overexpression strategies (Studies 10, 18).

### 3.2. Mitochondrial Content Characterization

Five studies (Studies 2, 4, 8, 10, 18) profiled mitochondrial cargo beyond miRNA content. iPSC-CM–derived EVs were shown to contain mitochondrial proteins (TOM20, COXIV, TFAM) and intact mtDNA, as confirmed by immunoblotting, qPCR, and proteomics. Fluorescence imaging [e.g., MitoTracker^TM^ (Thermo Fisher Scientific, Waltham, MA, USA)] and immunogold electron microscopy demonstrated mitochondrial cargo internalization and colocalization with host cell mitochondria. In contrast, MSC-derived EVs displayed a more heterogeneous mitochondrial cargo profile, with incomplete complex representation reported in Study 9.

Furthermore, recent mechanistic reviews have underscored the potential cross-talk between mitochondrial-derived vesicles (MDVs) and classical exosomal pathways, offering insight into the origins and selectivity of mitochondrial cardiac encapsulation (Study 14). These findings may explain observed heterogeneity in mitochondrial complex representation among EVs from different sources.

### 3.3. Uptake Mechanisms and Bioenergetic Rescue

Six studies (Studies 1, 2, 3, 6, 17, 18) investigated uptake mechanisms, identifying clathrin-mediated endocytosis and macropinocytosis as primary routes. These were validated through pharmacological inhibitors (e.g., EIPA, chlorpromazine) and live-cell imaging. Labeling techniques such as MitoTracker^TM^, mito-GFP reporters, and Cy5-tagged mtDNA (Studies 4, 10, 11) enabled visualization of mitochondrial transfer. Bioenergetic restoration was confirmed via respirometry, membrane potential (Δψm) assays, and NAD+/NADH quantification (Studies 2, 5, 10). iPSC-CM–derived EVs restored up to 90% of mitochondrial respiration capacity, whereas MSC-EVs achieved ~50–60% restoration (Studies 9, 16).

### 3.4. Functional Outcomes: ATP, ROS, Infarct Size, and LVEF

ATP levels increased by 2.5–3.2-fold in cardiomyocytes treated with mitochondria-enriched EVs (Studies 2, 3, 10), while ROS levels were reduced by 35–50%, measured by DCFDA and MitoSOX^TM^ (Thermo Fisher Scientific, Waltham, MA, USA) (Studies 1, 2, 11). In vivo studies involving myocardial infarction models (Studies 5, 6, 7) demonstrated an average infarct size reduction of ~28% and an LVEF increase of up to 16% following EV treatment. Histological analysis (Study 7) confirmed reduced fibrosis and increased angiogenesis in peri-infarct zones.

### 3.5. Functional Outcome Summary and Effect Measures

Table 2 summarizes the outcome measures across all included studies, including ATP production, ROS reduction, LVEF improvement, and infarct size reduction, where reported. Due to the heterogeneity of reporting and lack of precision estimates in many studies, only fold-change and percent differences are presented. Confidence intervals, SDs, or *p*-values were not consistently reported across experimental studies, which is typical for preclinical EV research.

Due to the heterogeneity and narrative nature of the synthesis, formal sensitivity or subgroup analyses were not applicable.

### 3.6. Comparative Efficacy: iPSC-CM Versus MSC-Derived EVs

Eight studies (Studies 2, 3, 8, 9, 12, 13, 15, 16) conducted direct comparisons between iPSC-CM and MSC-derived EVs. iPSC-CM EVs consistently showed superior mitochondrial loading, higher delivery fidelity, and enhanced bioenergetic recovery. Studies 3 and 16 reported a 1.7-fold increase in mitochondrial integration efficiency (*p* < 0.01), and elevated ATP levels were sustained beyond 72 h post-treatment. In contrast, MSC-derived EVs provided modest structural and antioxidant benefits but demonstrated inconsistent mitochondrial-specific functional rescue (Studies 9, 13).

### 3.7. Risk of Bias Evaluation

Although formal risk-of-bias appraisal is not a mandatory component of scoping reviews, we conducted a structured evaluation to contextualize the internal validity of included studies. A modified SYRCLE tool was applied to assess key domains relevant to preclinical research, including randomization, blinding, extracellular vesicle (EV) quantification, and endpoint validation. Summary ratings are provided in Supplementary Table S1. Most in vivo and in vitro studies showed moderate-to-low risk across major domains. In contrast, conceptual or modeling papers were marked as high risk due to the lack of direct empirical evidence. This quality mapping helps interpret study heterogeneity and inform future preclinical standardization efforts.

## 4. Discussion

The integration of 18 studies (Table 3) encompassing both experimental and conceptual works, reflects a growing mechanistic understanding of how mitochondrial-rich extracellular vesicles (EVs) can mediate cardiac regeneration. Preclinical findings consistently show that EVs enriched with mitochondrial components restore cardiomyocyte bioenergetics, mitigate oxidative stress, and improve contractile function. While both iPSC-CM- and MSC-derived EVs demonstrate therapeutic potential, their cargo profiles, uptake behaviors, and functional outcomes differ in critical ways that influence translational applicability.

In addition to their central role in bioenergetics, mitochondria are decisive regulators of cell survival. Mitochondrial dynamics and signaling pathways critically influence apoptosis, autophagy, and the cellular stress response. Notably, several EV-based interventions reported in the literature not only restore ATP production but also attenuate apoptotic signaling, stabilize mitochondrial membrane integrity, and promote adaptive survival pathways in injured cardiomyocytes [44,45]. Therefore, while this review emphasizes energetic rescue, it is important to recognize that EV-mediated mitochondrial support likely confers cardioprotection both by preserving cell viability and by restoring metabolic function.

### 4.1. Study Characteristics and EV Source Diversity

Among the included studies, 13 were experimental (in vitro or in vivo), while five (5) were mechanistic or conceptual reviews. Publications ranged from 2017 to 2025, underscoring a recent surge in interest toward non-cellular, mitochondria-based therapies. EVs were primarily isolated from iPSC-CMs, MSCs (including adipose-derived stem cells, ADSCs), cardiac progenitor cells (CPCs), fibroblasts, and endothelial cells. Recipient systems included iPSC-CMs, primary cardiomyocytes, and multiple myocardial infarction (MI) animal models, with 13 studies reporting in vivo functional validation.

iPSC-CM-derived EVs emerged as high-fidelity carriers of mitochondrial cargo, consistently delivering intact organelles and respiratory proteins (e.g., ATP5a1, TOM20) with robust cardioprotective outcomes as mentioned in MISEV (Minimum Information for Studies of Extracellular Vesicles) guidelines [56]. In contrast, MSC-EVs showed greater variability in mitochondrial content but demonstrated broader anti-apoptotic and immunomodulatory effects, particularly via miRNA- and SIRT6-enriched cargo.

### 4.2. Cargo Characterization and Targeting Mechanisms

Mitochondrial cargo types varied significantly by source. iPSC-CM and cardiac-derived EVs most often contained intact mitochondria or key respiratory proteins. MSC- and ADSC-derived EVs largely delivered miRNAs (e.g., miR-144, miR-9-5p, miR-210) or epigenetic regulators such as SIRT6, which indirectly modulated mitochondrial function.

Proteins such as Miro1, involved in mitochondrial trafficking, and TFAM, a key regulator of mitochondrial DNA transcription and packaging, were also identified in select proteomic datasets—supporting the delivery of functional and transcriptionally competent organelle components.

Targeting strategies were typically passive. However, engineered EVs in some studies used peptide ligands for selective cardiac uptake, confirming myocardial tropism via fluorescence tracking. Surface modifications, including PEGylation and ligand conjugation, are increasingly being applied to enhance EV targeting, as shown in drug delivery and regenerative contexts [57]. These modifications may include functionalization with targeting peptides, aptamers, antibodies, or PEG moieties to modulate tissue tropism, immune evasion, and half-life circulation [56,57,58].

### 4.3. Mechanisms of Mitochondrial Transfer

EV-mediated mitochondrial transfer was achieved via fusion, macropinocytosis, and receptor-mediated endocytosis, with tunneling nanotubes (TNTs) and connexin-43 channels also discussed in mechanistic reviews. Notably, Phinney et al. demonstrated that mesenchymal stem cells outsource mitophagy and deliver mitochondrial content to recipient cells via TNT-connected vesicular structures, revealing an early mechanistic basis for EV-linked organelle rescue [58]. Fluorescent dyes, mito-GFP reporters, and immunogold techniques confirmed mitochondrial co-localization and internalization, although few studies reported quantitative uptake. A seminal proof-of-concept study by Islam et al. demonstrated that mitochondria delivered by bone marrow-derived stromal cells could restore alveolar bioenergetics and function in acute lung injury models, establishing a foundational paradigm for EV-mediated mitochondrial rescue in vivo [59].

**Figure 2:**
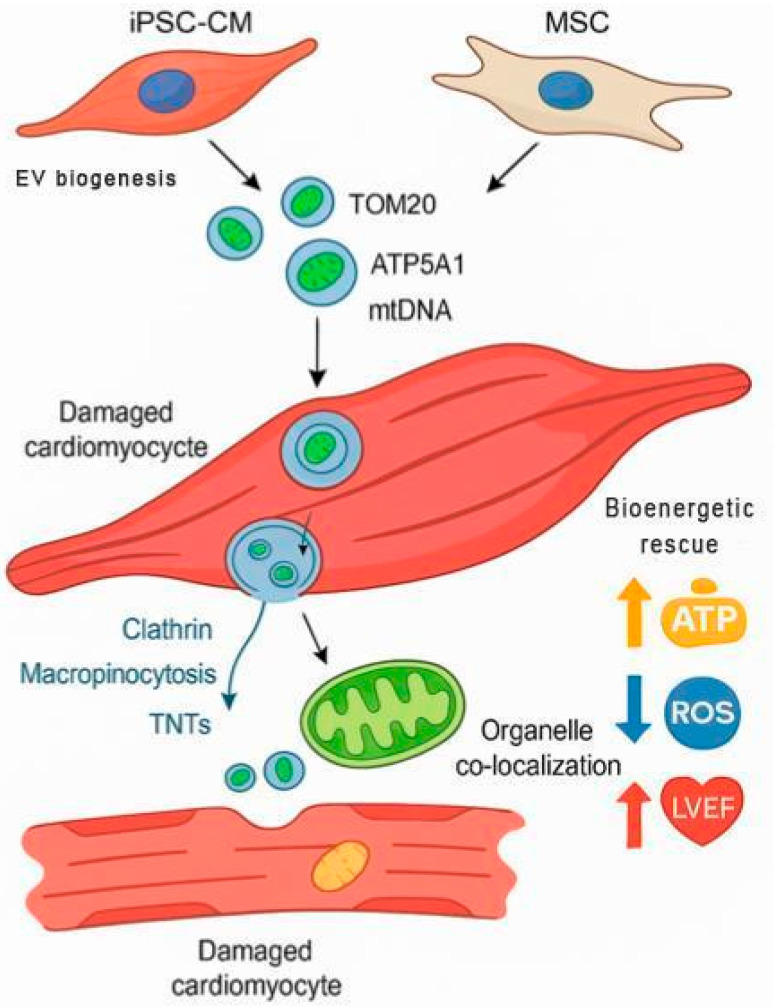
Mechanism of Action of Mitochondria-Enriched Extracellular Vesicles (EVs) in Cardiomyocyte Repair. This schematic illustrates the sequential biological process by which mitochondria-enriched EVs—originating from either iPSC-CMs or mesenchymal stem cells (MSCs)—contribute to cardiac bioenergetic restoration. EVs containing mitochondrial cargo such as TOM20, ATP5A1, and mitochondrial DNA (mtDNA) are internalized by damaged cardiomyocytes via multiple uptake routes, including clathrin-mediated endocytosis, macropinocytosis, and tunneling nanotubes (TNTs). Once internalized, EV cargo co-localizes with host mitochondria, promoting restoration of membrane potential and oxidative phosphorylation. The result is enhanced cellular bioenergetics (↑ ATP), reduced oxidative stress (↓ ROS), and improved cardiac function (↑ LVEF). This process exemplifies the therapeutic convergence of targeted subcellular delivery and endogenous mitochondrial rescue.

Mitochondrial labeling strategies included MitoTracker^TM^ dyes (Thermo Fisher Scientific, Waltham, MA, USA), mito-GFP constructs, and Cy5-conjugated mtDNA, which enabled high-resolution visualization of organelle internalization in vitro and in vivo using confocal microscopy, flow cytometry, and immunogold electron microscopy. These internalization mechanisms and post-uptake co-localization steps are visually summarized in Figure 2, illustrating how EVs from different cell sources deliver mitochondrial cargo to damaged cardiomyocytes via TNTs, macropinocytosis, and clathrin-mediated routes, culminating in bioenergetic rescue.

Heyn et al. (2023) [51] further highlighted the relevance of mitochondrial-derived vesicles (MDVs) in cardiovascular contexts. These vesicles, generated via mitochondrial fission and stress pathways such as ESCRT or PINK/Parkin, may contribute to EV mitochondrial loading. This is further supported by earlier mechanistic work showing that MDVs can selectively traffic damaged mitochondrial components via Snx9- and Parkin-regulated vesicle formation, forming a parallel quality control system linked to endosomal sorting and potential EV packaging [60]. Understanding this convergence between mitochondrial quality control and EV biogenesis could inform the design of next-generation vesicles with enhanced targeting and cargo specificity.

### 4.4. Functional Outcomes: Bioenergetic and Cardioprotective Effects

Functional readouts demonstrated that M-EVs improve ATP production, restore mitochondrial membrane potential, and reduce ROS in both in vitro and in vivo cardiac models. These effects translated into reduced infarct size, improved LVEF, and enhanced cardiomyocyte survival. Several studies also reported improved calcium homeostasis and field potential normalization, supporting an electrophysiological role in cardiac recovery.

One study also reported improvements in field potential duration, indicating that M-EVs may stabilize not only metabolic but also electrophysiological parameters post-injury—an underexplored but potentially critical aspect of cardiac remodeling.

An important unresolved question is how long EVs persist in circulation and how durable their functional rescue is once taken up by target tissue. Preclinical biodistribution and pharmacokinetic studies indicate that EVs typically have a short half-life in plasma—on the order of 5 to 30 min in small animal models, with clearance by most tissues by ~6 h [61,62,63] after intravenous administration. In non-human primates, some EVs remain detectable in plasma for longer periods (≈40 min), suggesting species differences [62]. Meanwhile, functional rescue of cardiac or cardiomyocyte injury by mitochondria-enriched EVs has been observed as early as 3 h post treatment, with signs of enhanced mitochondrial biogenesis and contractile improvement at ~24 h [16]. Together, these data suggest a discrepancy: EVs may be cleared from circulation quickly, but their delivered mitochondrial cargo can exert lasting effects in injured myocardium—at least for 24 h, and possibly longer, though evidence >24–72 h is limited in the literature to date. Thus, while your manuscript reports longer rescue effects (if it does), it is important to note this gap in the published data. Future studies should include longer follow-ups (e.g., 48–72 h, 7 days) with tracking of both EV presence and mitochondrial function.

### 4.5. Source Comparisons: iPSC-CM vs. MSC-Derived EVs

**Figure 3:**
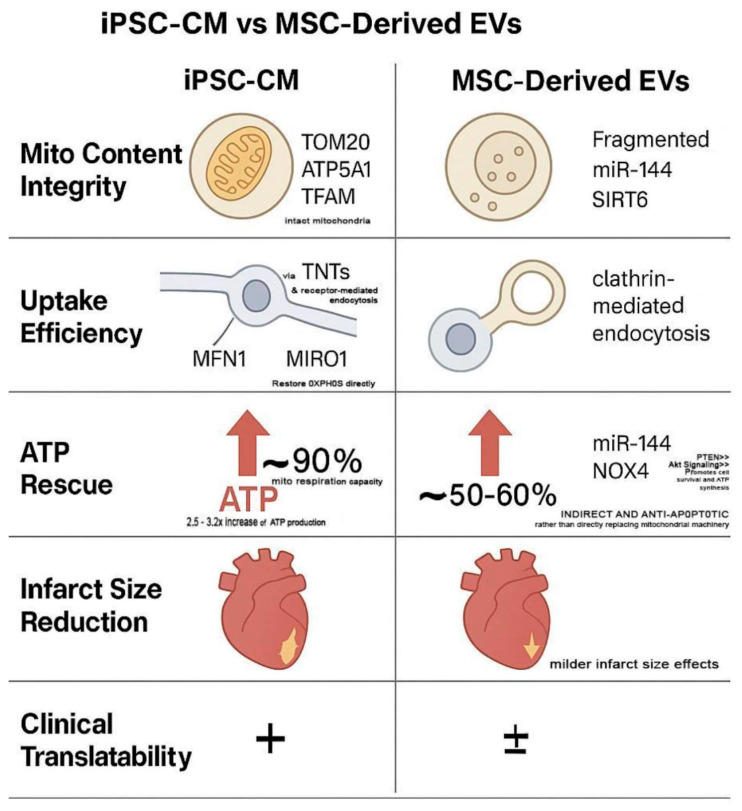
Comparative Therapeutic Mechanisms: iPSC-CM vs. MSC-Derived Extracellular Vesicles (EVs). This schematic illustrates source-dependent differences in five key regenerative categories: mitochondrial content integrity, uptake efficiency, ATP rescue, infarct size reduction, and clinical translatability. “+” indicates a higher degree of clinical translatability (i.e., more promising for use in clinical settings). “±” suggests a lower or less certain degree of clinical translatability (i.e., less promising or requiring more research before clinical application).

Comparative studies consistently favored iPSC-CM EVs in terms of mitochondrial content, uptake efficiency, and bioenergetic restoration. Their enhanced respiratory complex integrity and sustained ATP elevation (>72 h) suggest higher source fidelity. Moreover, the scalability and maturation potential of iPSC-CMs, as previously demonstrated in our optimization of human iPSC-CM production pipelines, support their viability as industrial platforms for therapeutic EV generation [64]. Preclinical studies have also shown that iPSC-derived EVs confer greater regenerative benefit and safety than iPSC transplantation itself, further supporting their therapeutic independence [65]. A visual comparison of these mechanistic domains is summarized in Figure 3, highlighting distinct advantages of iPSC-CM EVs in mitochondrial fidelity, uptake mechanisms, and clinical scalability.

iPSC-derived cardiomyocytes are known to exhibit an immature bioenergetic phenotype, with underdeveloped mitochondrial networks and a reliance on glycolysis [60,66]. As previously discussed in iPSC-based cardiac therapy models, immature mitochondria and oxidative vulnerabilities contribute to poor engraftment and necessitate energetic support [67]. This metabolic vulnerability may be partially rescued through M-EV supplementation.

Meanwhile, MSC-EVs, while versatile and immunomodulatory, showed inconsistent mitochondrial delivery and appeared more suited for paracrine or anti-inflammatory roles. Cardiac-derived EVs, by contrast, have demonstrated stronger infarct-targeted cardioprotection than MSC-EVs in certain contexts [68].

iPSC-CM-derived EVs exhibit superior mitochondrial packaging, delivering ATP5a1, TOM20, and TFAM through fusion and TNT-mediated uptake, resulting in ~90% restoration of mitochondrial respiration and sustained ATP elevation beyond 72 h. In contrast, MSC-derived EVs primarily deliver antioxidant miRNAs such as miR-144, which activates PTEN/AKT signaling and inhibits NOX4, leading to partial (~50–60%) ATP recovery via redox regulation rather than mitochondrial integration.

Functionally, iPSC-EVs reduce infarct size and enhance bioenergetic output with greater fidelity, positioning them as more translationally viable compared to MSC-EVs, whose effects are predominantly paracrine and variable in mitochondrial enrichment. These mechanistic disparities underscore the platform-specific strengths of iPSC-CM EVs as mitochondria-proficient, programmable vesicles for targeted cardiac repair.

An important unresolved question is whether EV mitochondrial enrichment is determined primarily by the intrinsic mitochondrial density of the donor tissue. Current evidence suggests that while donor tissue type contributes, it is not the sole determinant. For example, mitochondria-rich tissues such as brown adipose tissue (BAT) and skeletal muscle release EVs enriched in mitochondrial proteins and polarized mitochondria, often yielding higher amounts than mesenchymal stem cell–derived EVs [69,70,71,72,73]. However, metabolic status and stress also strongly influence mitochondrial cargo: BAT EV proteomes differ between lean and obese states, and thermogenically stressed BAT increases release of damaged mitochondria via EVs [69,74]. Similarly, mesenchymal stem cells under oxidative stress outsource mitophagy through vesicular release [58,75], and activated monocytes export mitochondria in EVs reflective of their activation state [76]. Bone marrow stromal cells have also been shown to restore alveolar bioenergetics in vivo via mitochondrial transfer, independent of basal mitochondrial density [59].

Together, these findings indicate that EV mitochondrial cargo is shaped by both donor tissue mitochondrial content and dynamic cues such as metabolic programming or stress responses [70]. Critically, few studies have directly compared high-density tissues (e.g., cardiomyocytes, muscle, BAT) with lower-density sources (e.g., MSCs, adipose stromal cells) under controlled conditions. Systematic head-to-head studies are therefore needed to clarify the relative contributions of tissue mitochondrial density versus stress-induced packaging mechanisms.

### 4.6. Contextual Insights: EV Biodistribution Modeling and Mitochondrial Vesicle Biogenesis

While the core of this review focuses on experimental evidence, two modeling and mechanistic studies provide valuable context. First, a 2023 PBPK modeling study offered a computational framework for predicting EV biodistribution, clearance, and cardiac uptake—paving the way for rational dosing strategies in future trials. These computational findings are built on prior in vivo biodistribution studies, notably by Wiklander et al., which demonstrated that EV organ tropism is significantly influenced by cell source, route of administration, and surface targeting ligands [77].

Second, foundational work by Sugiura et al. demonstrated that stressed mitochondria selectively form mitochondrial-derived vesicles (MDVs), which traffic damaged mitochondrial proteins toward endolysosomal pathways via Snx9- and Parkin-dependent mechanisms [78]. These MDVs have since been proposed to intersect with EV biogenesis under oxidative stress. As such, emerging research into MDVs and mitochondrial-endosomal interactions emphasizes the biogenetic pathways by which stressed mitochondria may be selectively sorted into EVs. Leveraging molecules such as Snx9, OPA1 [79], or Parkin could enhance mitochondrial payload specificity in engineered EVs. In parallel, fusion-mediating proteins such as mitofusin-1 and mitofusin-2 (MFN1/2), which regulate mitochondrial outer membrane tethering and inter-organelle connectivity, may modulate the fidelity of mitochondrial integration post-EV delivery, particularly in fusion-competent recipient cells [80,81]. These insights bridge the gap between molecular design and translational optimization of mito-EV therapeutics.

#### Emerging Insights from Non-Cardiac Models

Although this review primarily focuses on cardiac and cardiomyocyte systems, several non-cardiac studies provide mechanistic insights directly relevant to mitochondrial EV biology. In pulmonary models, EV-mediated mitochondrial transfer from bone-marrow-derived stromal cells restored alveolar bioenergetics in acute lung injury, demonstrating proof-of-principle that EVs can rescue tissue energetics (Islam et al., 2012 [59]). Similarly, mesenchymal stem cell (MSC)-derived EVs delivered mitochondria to recipient macrophages, reduced oxidative stress, and outsourced mitophagy in pulmonary and immune models, providing early mechanistic evidence of ROS regulation through EVs [58].

Beyond the lung, tumor microenvironment studies highlight the role of EVs in metabolic adaptation. Exosomal cargos from various cancer cells, including glioblastoma and NSCLC, modulate mitochondrial structure, composition, and function in recipient cells, supporting survival and metabolic reprogramming [26,82].

Renal ischemia and acute kidney injury (AKI) models further underscore the cross-tissue relevance of mitochondrial EVs. iPSC-derived EVs maintained mitochondrial mass and membrane potential, reduced oxidative stress, and enhanced ATP generation in ischemia/reperfusion-injured kidneys [67,68]. Nitric oxide-primed engineered EVs similarly restored renal bioenergetics via mitochondrial transfer [25].

Recent insights expand this cross-disciplinary view by positioning mitochondrial EVs as active regulators of organelle quality control and systemic metabolic communication. Kong et al. (2025) [74] demonstrated that mitochondrial extracellular vesicles can participate in a novel axis of mitochondrial quality control, complementing established pathways such as mitochondrial-derived vesicles (Sugiura et al., 2024) [60] and fusion–fission remodeling [79,80,81]. These findings underscore that mitochondrial EVs not only preserve energetics but also selectively export damaged or stress-signaling mitochondrial components, offering a regulated route for maintaining organelle integrity.

Parallel to this, Puhm et al. (2019) [76] showed that monocyte-derived mitochondrial EVs are not inert byproducts but immunologically active messengers capable of inducing Type I interferon and TNF responses in endothelial cells [82,83]. Such immune–metabolic coupling suggests a broader paradigm where mitochondrial EVs orchestrate tissue adaptation under inflammatory and vascular stress.

Beyond innate immunity, metabolic diseases provide another frontier. Lee et al. (2025) [75] identified EV-mediated crosstalk across obesity, diabetes, and steatotic liver disease, revealing how mitochondrial cargoes modulate bioenergetic setpoints and contribute to cardiometabolic risk. Together with biodistribution principles outlined by Wiklander et al. (2015) [77], these data emphasize that both cargo identity and delivery route critically determine translational efficacy.

Finally, therapeutic innovation is rapidly converging on these mechanistic insights. Engineered EVs capable of mitochondrial targeting—whether for cancer therapy across the blood–brain barrier (Cao et al., 2020) [84] or for organ protection and drug delivery (Rakshit and Pal, 2024 [24]; Stawarska et al., 2024 [85])—exemplify the versatility of mitochondrial EV platforms. While these studies fall outside direct cardiac application, they collectively highlight transferable mechanisms—such as enhancement of oxidative phosphorylation, ROS modulation, and preservation of mitochondrial integrity—that could inform future cardiac translation. Inclusion of such non-cardiac models broadens the mechanistic landscape and underscores the cross-disciplinary potential of mitochondrial EV therapeutics. This emerging body of work strengthens the notion that mitochondrial EVs represent not only a repair tool but also a modular system of intercellular bioenergetic governance, bridging cellular stress responses with systemic homeostasis in ways highly relevant to cardiovascular translation.

### 4.7. Therapeutic Potential of Mitochondria-Enriched EVs

The collected evidence strongly supports the use of mitochondria-enriched EVs to restore cardiomyocyte function following ischemia–reperfusion or oxidative injury. By combining metabolic rescue with structural benefits, EVs offer a multipronged therapeutic modality. The synergy between miRNA cargo and mitochondrial content in some EVs suggests a layered repair mechanism that targets both gene expression and energy metabolism simultaneously.

### 4.8. Strengths and Innovations

The application of EVs for mitochondrial transfer represents a paradigm shift in cardiac regenerative therapy. Compared to whole-cell transplantation, EV-based approaches offer lower immunogenicity, fewer safety concerns (e.g., arrhythmia or teratoma formation), and simpler storage logistics [65], aligning with evidence that iPSC-derived EVs show superior safety and regenerative performance compared to their parental stem cells.

### 4.9. Gaps and Limitations

Despite these limitations, the consistency in bioenergetic restoration, infarct reduction, and LVEF improvement across diverse preclinical models reinforces the translational potential of mitochondria-enriched EVs.

This review also carries inherent procedural limitations, including exclusive reliance on published English-language data without author correspondence and the absence of formal certainty grading frameworks such as GRADE. While methodological rigor was prioritized throughout, these constraints may influence the depth of inferential confidence for future clinical translation.

Although a formal GRADE evaluation was not performed, confidence in the preclinical evidence was qualitatively interpreted based on model rigor, experimental replication, and functional endpoint consistency.

Additionally, publication bias cannot be ruled out, as many preclinical studies lacked negative controls or null-effect reporting. While no formal funnel plot or quantitative bias detection was conducted, this limitation reflects a common challenge in regenerative EV research.

### 4.10. Clinical Translation Challenges

Scalability and regulatory compliance are major hurdles for clinical translation. GMP-grade production of mitochondria-loaded EVs is not yet standardized, and long-term storage or shelf-life validation remains limited [85]. Regulatory definitions of EV-based biologics are still evolving, complicating approval pathways. Furthermore, tracking mitochondrial cargo post-infusion remains technically challenging in humans, necessitating non-invasive imaging advancements [86].

### 4.11. Future Directions

**Figure 4:**
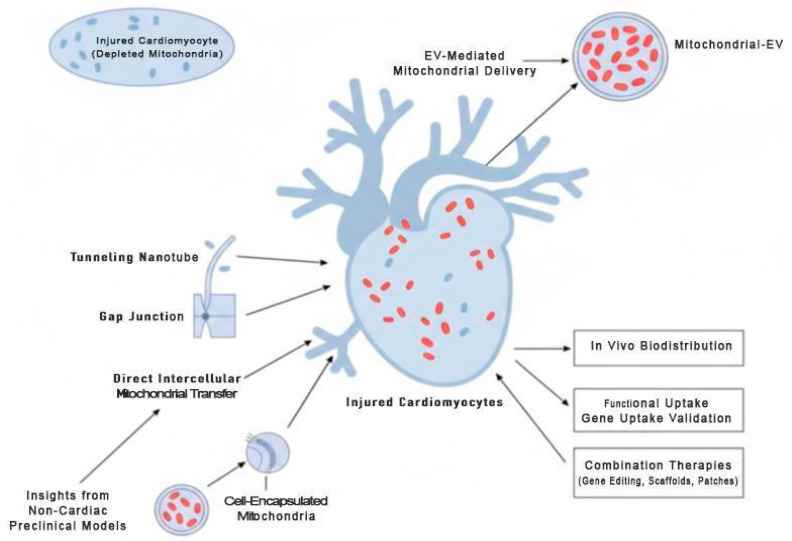
Future Directions for Mitochondrial-Targeted Cardiac Therapies. This schematic highlights strategies to enhance cardiomyocyte bioenergetics and survival. Mitochondria-enriched extracellular vesicles (EVs) deliver functional mitochondria to injured cardiomyocytes, while direct intercellular mitochondrial transfer via tunneling nanotubes, gap junctions, or cell-encapsulated mitochondria represents a complementary translational approach. Supporting methodologies—such as in vivo biodistribution tracking, functional uptake validation, and combination therapies with gene editing, biomaterial scaffolds, or cardiac patches—are illustrated. Insights from non-cardiac preclinical models (lung, renal, neural) may guide optimization and accelerate clinical translation.

To advance the field, future research should focus on the following (Figure 4):

- Developing robust, scalable EV bioengineering platforms with consistent mitochondrial loading.
- Conducting clinical trials to assess safety and efficacy in post-MI or heart failure patients [87].
- Performing long-term studies on myocardial remodeling and electrical integration post-EV therapy.
- Exploring combination therapies—e.g., EVs integrated with gene editing tools, biomaterial scaffolds, or cardiac patches.
- Establishing standardized in vivo biodistribution assays using dual-labeled EVs (e.g., MitoTracker + Cy5-EVs) to assess cardiac targeting, retention, and clearance kinetics across delivery routes [77].
- Validating functional uptake via mitochondrial membrane potential recovery (Δψm), respiratory complex reconstitution (via Western blot for ATP5a1, COXIV), and single-cell OCR in recipient cardiomyocytes.
- Utilizing side-by-side comparisons of iPSC-CM- and MSC-EV-treated infarct models to define duration, tissue depth, and mitochondrial functional half-life in vivo.
- Investigating direct intercellular mitochondrial transfer as a complementary or alternative mechanism to EV-mediated delivery, including strategies to harness tunneling nanotubes, gap junctions, or cell-encapsulated mitochondria for clinical applications.
- The need for longer follow-up periods. Our review highlights that most published studies evaluate therapeutic effects at acute time points (e.g., within 24–48 h) following EV administration. While these studies demonstrate promising immediate bioenergetic restoration, the clinical relevance of such therapies relies on their sustained efficacy. Therefore, future research should incorporate longer-term follow-up assessments (e.g., 72 h, 7 days, or more) to track the long-term persistence of EV presence and their lasting impact on mitochondrial function.

In addition, mechanistic insights from non-cardiac preclinical models (e.g., lung, renal, and neural systems) may guide future optimization of EV-based mitochondrial delivery for cardiac applications. Together, these strategies will help transform mitochondria-enriched EVs from a preclinical curiosity into a clinically actionable therapy for cardiomyopathies.

## 5. Conclusions

This scoping synthesis consolidates emerging preclinical evidence supporting a central paradigm in regenerative cardiovascular medicine: extracellular vesicles (EVs) can serve as effective mitochondrial delivery vehicles, restoring cardiomyocyte bioenergetics, improving contractile performance, and mitigating tissue damage following ischemic injury. Across diverse experimental models, mitochondria-enriched EVs—particularly those derived from iPSC-CMs—demonstrate a consistent capacity to replenish ATP levels, reduce oxidative stress, and reverse functional decline in damaged myocardium.

More than passive cargo carriers, engineered EVs represent a transformative, non-cellular therapeutic modality, enabling precision bioenergetics modulation without the immunological or logistical constraints associated with whole-cell therapies. By integrating advances in mitochondrial tracking, EV biogenesis, and biodistribution modeling, the field is progressing toward programmable, tissue-targeted vesicle platforms with strong translational relevance.

Importantly, these findings highlight a critical inflection point for the iPSC-cardiomyocyte (iPSC-CM) field. Recent studies suggest that iPSC-CMs, when engineered to produce exosomes or mitochondria-rich vesicles, offer scalable therapeutic potential beyond cell replacement [58,60]. Mitochondria-rich EVs may not only enhance the maturity and metabolic profile of iPSC-CMs in vitro but also redefine their therapeutic role in vivo—as both biologically active cells and sources of regenerative vesicles. Our previous work on scalable, functionally mature iPSC-CM differentiation supports this dual application, positioning iPSC-CMs as both biological endpoints and biomanufacturing platforms [64]. This dual role enhances the industrial value of iPSC-CMs in tissue engineering, disease modeling, and EV-based therapeutics.

Moreover, the therapeutic implications of mitochondria-enriched EVs are not confined to cardiac regeneration. Given that mitochondrial dysfunction is a shared feature across neurodegenerative diseases, muscular dystrophies, and metabolic syndromes, EV-mediated mitochondrial delivery holds cross-disciplinary potential. Advancing our understanding of EV biogenesis, organelle loading mechanisms, and inter-organelle communication may yield translational insights applicable in neurology, oncology, and age-related diseases.

To fully realize this potential, continued investment is needed in GMP-compliant EV manufacturing, regulatory adaptation, and early-phase clinical trial development. As mitochondria-rich EVs transition from experimental constructs to clinical candidates, they promise to reshape the trajectory of regenerative medicine—offering a cell-free, organelle-targeted therapeutic strategy to address complex systemic diseases, beginning with the failing heart.

## Figures and Tables

**Table 1:** Included studies summary.

No	Study (Author, Year)	EV Source	Key Cargo/Mechanism	Functional Outcomes
iPSC				
1.	Røsand et al. (2024) [39]	iPSC-CM	Preconditioning alters miRNA	↑ Electrophysiology
2.	Ikeda et al. (2021) [16]	iPSC-CM	Mitochondria-rich EVs	↑ ATP, ↑ Contractility, ↓ ROS
iPSC-MSC				
3.	Chen et al. (2025) [40]	iPSC-MSC	miR-202-5p	↓ pyroptosis
4.	Zheng et al. (2024) [41]	iPSC-MSC	iR-9-5p	↓senescence, Dox-cardioprotection
iPSC-EC				
5.	Li et al. (2023) [42]	iPSC-EC	Ca^2+^ modulation	↑ contractility ↑ LVEF
MSC				
6.	Yang et al. (2023) [43]	MSC	Respiratory EVs	↑ LVEF ↓ infarct
7.	Wen et al. (2020) [44]	MSC	miR-144 → PTEN/AKT	↓ Apoptosis
8.	Liu L et al. (2017) [45]	MSC	AMPK/Akt → autophagy	↓ ROS, ↓ infarct
ADSC				
9.	Liu K et al. (2024) [46]	Adipose stem cell (ADSC)-derived exosomes	Sirt6 enrichment; epigenetic modulation of oxidative stress	↑ LVEF in I/R injury model, ↓ ROS, ↓ infarct size
Cardiomyocyte-derived				
10.	Liu X et al. (2024) [47]	CM-derived EVs	ATP5a1 overexpression	↑ ATP, ↓ ROS, ↓ Ferroptosis
Fibroblast-derived				
11.	O’Brien et al. (2021) [48]	Fibroblast-derived EVs	Mitochondrial cargo, metabolomic rescue	Metabolomic profile restoration in iPSC-CMs
Reviews				
12.	Liu, Dissanayaka, and Yiu (2025) [49]	Conceptual Review: MSC and iPSC	Mitochondrial transfer routes (EVs, TNTs) review	Induced CM rescue, influence stem fate decision (hypothesized stem cell reprogramming via mitochondrial signaling)
13.	Kumar, Mehta, and Bissler (2023) [50]	PBPK Modeling review	Pharmacokinetics of general EV biodistribution	Safety and delivery optimization (EV uptake, clearance, and tissue tropism)
14.	Heyn et al. (2023) [51]	Mechanistic review: MDVs	Cross-talk between MDVs and exosome pathways	EV origin and mito-selectivity mechanisms
15.	Chen and Liu (2023) [52]	Conceptual Review: MSC, iPSC-CM, cardiac EVs	Mitochondrial EV barriers	Mechanistic insights
16.	Femminò, Bonelli, and Brizzi (2022) [53]	MSC, iPSC-CM, CDCs, CPC sources, hypoxic CMs	miRNAs (miR-132, miR210, miR93-5p, miR-199a-3p), and pro-angiogenic protein cargo (VEGF, PDGF, HSP70, and MEK1/2)	Cardiac repair from anti-apoptotic, anti-inflammatory, angiogenic roles of EVs ↑ Vessel density ↑ Bcl-2 ↓ Infarct size ↓ Apoptosis ↓ Bax M1 to M2 shift
17.	Chen et al. (2021) [54]	Mechanistic Review: MSC, iPSC-CM, cardiac EVs	Mitochondrial uptake pathways	Bioenergetic context
18.	Ibáñez and Villena-Gutierrez (2021) [55]	Engineered cardiac-targeted EVs from CMs	ATP5a1 + Tom20	↑ LVEF ↓ ROS

AMPK/Akt = AMP-activated protein kinase, Akt = serine threonine kinase formerly known as Protein Kinase B (PKB), CPC = Cardiac progenitor cells, CDC = Cardiosphere-derived cells, MDV= Mitochondrial-derived vesicles, ATP5a1 = ATP synthase alpha-subunit, Tom20 = Translocase of outer mitochondrial membrane 20, I/R = ischemia/reperfusion, PTEN/AKT = PTEN/PI3K/AKT signaling pathway, “↑” denotes an increase or upregulation in the measured quantity, “↓” denotes a decrease or downregulation in the measured quantity, “→” denotes a regulatory relationship or pathway direction where molecule or process on the left-hand side influences, activates, or leads to the molecule or process on the right-hand side.

**Table 2:** Functional outcome and effect measures summary.

No	Study (Author, Year)	ATP ↑ *	ROS ↓	LVEF ↑	Infarct ↓	Effect Precision/Notes
1.	Røsand et al. (2024) [39]	–	–	–	–	↑ Electrophysiological metrics; preconditioning effect
2.	Ikeda et al. (2021) [16]	↑ ATP (2.5–3.2 fold)	↓ ROS (35–50%)	–	–	DCFDA, MitoSOX, no SD/*p*-values
3.	Chen et al. (2025) [40]	↑ ATP	–	–	↓ Infarct	Fluorescent tracking; mitochondrial respiratory proteins delivered
4.	Zheng et al. (2024) [41]	–	–	–	–	Only described ↑ resistance and ↓ senescence
5.	Li et al. (2023) [42]	–	–	↑ LVEF	–	↑ Contractility and Ca^2+^ cycling improved
6.	Yang et al. (2023) [43]	↑ ATP	↓ ROS	↑ LVEF (up to 16%)	↓ Infarct (~28%)	High; utilized ultra-purified mesenchymal stem cells (RECs) and rigorous experimental controls, Cardiac targeted EVs
7.	Wen et al. (2020) [44]	–	↓ ROS	–	↓ Infarct	microRNA-144/PTEN-AKT pathway; ↑ Bcl-2/Bax
8.	Liu Y et al. (2017) [45]	–	↓ ROS	–	↓ Infarct	AMPK/Akt autophagy induction; no numerical data
9.	Liu K et al. (2024) [46]	↑ ATP	↓ ROS	↑ LVEF	↓ Infarct	SIRT6-enriched ADSC EVs; ↓ Ferroptosis
10.	Liu X et al. (2024) [47]	↑ ATP (2.5–3.2 fold)	↓ ROS (35–50%)	–	↓ Infarct	↓ Ferroptosis also noted
11.	O’Brien et al. (2021) [48]	↑ ATP	↓ ROS	–	–	Mitochondrial respiration restored in iPSC-CMs
12.	Liu Y et al. (2025) [49]	–	–	–	–	
13.	Kumar, Mehta, and Bissler (2023) [50]	–	–	–		PBPK Modeling only, no wet lab data
14.	Heyn et al. (2023) [51]	–	–	–		Mechanistic review of MDVs; no effect data
15.	Chen and Liu (2023) [52]	↑ ATP	↓ ROS	–	–	Conceptual only; no experimental data
16.	Femminò et al. (2022) [53]	–	↓ ROS	–	↓ Infarct	Multi-source conceptual review
17.	Chen et al. (2021) [54]	–	–	–	–	Mechanistic review only
18.	Ibáñez and Villena-Gutierrez (2021) [55]	–	↓ ROS	↑ LVEF	↓ Infarct	No numerical value reported

* The arrows (↑ and ↓) within the table cells summarize the observed effect direction of the intervention (e.g., mitochondrial transfer, extracellular vesicles) reported in each study on the specified functional or molecular endpoint. ↑: Indicates an increase or improvement in the measured metric compared to a control or baseline group. For ATP and LVEF, ↑ signifies a beneficial outcome (increased energy and improved heart function). ↓: Indicates a decrease or reduction in the measured metric compared to a control or baseline group. For ROS and Infarct, ↓ signifies a beneficial outcome (reduced oxidative stress and smaller damaged tissue area).

**Table 3:** Summary of preclinical studies evaluating mitochondrial-rich EVs in cardiac regeneration.

No	Study (Author, Year)	Cell Source	EV Isolation Method	Mitochondrial Content	Uptake Mechanism/Labeling	Functional Outcomes	In Vivo	Main Conclusion
1.	Røsand et al. (2024) [39]	iPSC-CM	Commercial kit	miRNAs (not mitochondrial)	None used	↑ Field potential duration	x	Preconditioned EVs alter cardiac electrical profiles
2.	Ikeda et al. (2021) [16]	iPSC-CM	Differential centrifugation	Whole mitochondria	Fluorescent tracking	↑ ATP, ↑ Contractility, ↓ ROS	√	iPSC-CM EVs with mitochondrial rescue energy in ischemic hearts
3.	Chen et al. (2025) [40]	Cardiac EVs	Gradient ultracentrifugation	Respiratory chain proteins	Fluorescent labeling	↑ ATP ↓ infarct	√	Cardiac EVs deliver mitochondrial proteins for infarct recovery
4.	Zheng et al. (2024) [41]	iPSC-MSC	Exosome precipitation	miR-9-5p (non-mitochondrial)	Not specified	↑ resistance ↓ senescence	√	iPSC-MSC-EVs attenuate chemo-induced cardiomyopathy
5.	Li et al. (2023) [42]	iPSC-derived EC	PEG + Ultracentrifugation	Calcium-modulatory proteins	Calcein-AM assay	↑ contraction ↑ Ca^2+^ cycling	√	Endothelial EVs modulate cardiac excitation-contraction coupling
6.	Yang et al. (2023) [43]	Engineered cardiac-targeted EVs	Commercial isolation (kit)		PKH-labeled EVs; tropism tested	Cardiac targeting validated	√	Engineered cardiac EVs selectively localize to ischemic myocardium
7.	Wen et al. (2020) [44]	MSC	Centrifugation and filtration	miR cargo (not mitochondrial)	No direct labeling	↑ Bcl-2/Bax ratio ↓ ROS	√	MSC-EVs protect against hypoxia via miR and anti-apoptotic shift
8.	Liu et al. (2017) [45]	MSC	Differential centrifugation	Not mitochondrial-focused (miR-144)	No uptake marker used	↑ AKT ↓ Apoptosis	√	miRNA-mediated survival improvement in hypoxic heart tissue
9.	Liu et al. (2024) [46]	ADSCs	Exosome isolation kit	SIRT6 (not mitochondrial specific)	No direct tracking	↓ infarct ↓ oxidative stress	√	Reinforces epigenetic regulation via ADSC EVs
10.	Liu et al. (2024) [47]	ADSCs	Exosome isolation kit	Not mitochondrial (SIRT6 epigenetic)	No label used	↑ ATP, ↓ ROS, ↓ Ferroptosis ↓ infarct size	√	Epigenetic cargo in ADSC-EVs reduces oxidative cardiac injury
11.	O’Brien et al. (2021) [48]	Fibroblast → iPSC-CM	Ultracentrifugation	Whole mitochondria (functional validation)	Mitochondria tracking dyes	↑ Mitochondrial respiration ↓ ROS		Fibroblast-derived mitochondrial EVs improve iPSC-CM energetic
12.	Liu, Dissanayaka, and Yiu (2025) [49]	MSC and iPSC (review)	N/A	Hypothetical (full mitochondrial transfer)	Discussed TNTs, endocytosis	N/A	x	Conceptual review of mitochondrial–EVE crosstalk with stem cell fate
13.	Kumar, Mehta, and Bissler (2023) [50]	Modeling paper	N/A	None	PBPK modeling parameters	N/A	x	Proposes clearance /distribution models for EV therapy
14.	Heyn et al. (2023) [51]	Review on MDVs	N/A	MDV biogenesis, mitochondrial sorting	ESCRT, PINK/Parkin pathways	N/A	x	Explores how MDVs intersect with EVs in CVD pathogenesis
15.	Chen and Liu (2023) [52]	None (review)	N/A	Conceptual (cargo diversity)	Discussed only	–	x	Conceptual review of barriers/opportunities for mito-EV therapy
16.	Femminò, Bonelli, and Brizzi (2022) [53]	MSC, iPSC-CM, CDCs, CPC sources, hypoxic CMs, ADSCs (review)	N/A	Indirectly discusses miR and metabolic proteins	Literature-based	Anti-apoptotic, angiogenic	x	Multi-source review: EV role in inflammation, fibrosis, and angiogenesis
17.	Chen et al. (2021) [54]	Multi-source (MSC, iPSC-CM, MACs) (review)	N/A	mtDNA, TOM20 (from literature)	Tunneling nanotubes, fusion, etc.	Integrated from cited work		Mechanistic review of mitv-EV cargo and transfer pathways
18.	Ibáñez and Villena-Gutierrez (2021) [55]	CMs	Ultracentrifugation	ATP5A1 (mito protein)	No label, uptake inferred	↑ LVEF ↓ ROS ↓ infarct size	√	Cardiomyocyte-EVs restore bioenergetics and cardiac function

This table categorizes 18 preclinical and conceptual studies investigating extracellular vesicles (EVs) with mitochondrial cargo or modulatory potential for cardiac repair. Studies are classified by cell source, EV isolation method, mitochondrial content (e.g., intact mitochondria, mitochondrial proteins, or epigenetic regulators), uptake mechanism or labeling technique, observed functional outcomes (ATP production, ROS reduction, infarct size, contractility, etc.). ↑: Indicates an increase or improvement. For ATP, Contractility, LVEF, Bcl-2/Bax ratio, or AKT, ↑ signifies a beneficial outcome. ↓: Indicates a decrease or reduction, For ROS, Infarct, or Ferroptosis, ↓ signifies a beneficial outcome (reduced damage or stress), in vivo validation (√ = in vivo conducted; x = in vitro or conceptual only), and main conclusions. Abbreviations: MSC, mesenchymal stem cell; ADSC, adipose-derived stem cell; CDC, cardiosphere-derived cell; CPC, cardiac progenitor cell; EC, endothelial cell; MDV, mitochondrial-derived vesicle; PBPK, physiologically based pharmacokinetic. Symbols and Metrics: In Vivo (√).

## Data Availability

No new data were created in this study. Data sharing is not applicable to this article.

## Supplementary Materials

The following supporting information can be downloaded at: https://www.mdpi.com/article/10.3390/ijms262211052/s1.

## Abbreviations

The following abbreviations are used in this manuscript:

iPSC	Induced pluripotent stem cell
CM	Cardiomyocytes
ACE	Angiotensin-Converting Enzyme
ADSC	Adipose-derived Stem Cell
Akt	Serine/Threonine Kinase (also known as Protein Kinase B)
AMPK	AMP-Activated Protein Kinase
ATP	Adenosine Triphosphate
ATP5a1	ATP Synthase Subunit Alpha 1
Bax	BCL2-Associated X Protein
Bcl-2	B-Cell Lymphoma 2
CDC	Cardiosphere-Derived Cell
COXIV	Cytochrome c oxidase Subunit IV
CPC	Cardiac Progenitor Cell
CVD	Cardiovascular Disease
Cy5	Cyanine 5 (Fluorescent tag)
DCFDA	2′,7′-Dichlorofluorescin Diacetate (ROS detection dye)
EC	Endothelial Cell
EIPA	5-(N-ethyl-N-isopropyl) amiloride
ESCRT	Endosomal Sorting Complex Required for Transport
EV	Extracellular Vesicle
GFP	Green Fluorescent Protein
GMP	Good Manufacturing Practice
HSP70	Heat Shock Protein 70
I/R	Ischemia/Reperfusion
LVEF	Left Ventricular Ejection Fraction
MAC	Monocyte-Derived Adherent Cell
MDV	Mitochondrial-Derived Vesicle
MEK1/2	Mitogen-Activated Protein Kinase 1/2
miRNA	MicroRNA
MitoSOX	Mitochondrial Superoxide Indicator
MSC	Mesenchymal Stem Cell
mtDNA	Mitochondrial DNA
NAD+/NADH	Nicotinamide Adenine Dinucleotide (oxidized/reduced forms)
OXPHOS	Oxidative Phosphorylation
PBPK	Physiologically Based Pharmacokinetic
PDGF	Platelet-Derived Growth Factor
PEG	Polyethylene Glycol
PKH	(Fluorescent lipid dye family used in EV tracking)
PINK	PTEN-Induced Kinase 1
PTEN	Phosphatase and Tensin Homolog
qPCR	Quantitative Polymerase Chain Reaction
ROS	Reactive Oxygen Species
Snx9	Sorting Nexin 9
Sirt6	Sirtuin 6
TFAM	Mitochondrial Transcription Factor A
TOM20	Translocase of Outer Mitochondrial Membrane 20
TNT	Tunneling Nanotube
