# Supplementary material for: Mitochondria-Enriched Extracellular Vesicles (EVs) for Cardiac Bioenergetics Restoration: A Scoping Review of Preclinical Mechanisms and Source-Specific Strategies
Source: Int J Mol Sci. 2025 Nov 15;26(22):11052. doi: 10.3390/ijms262211052 (PMC12652545; doi:10.3390/ijms262211052)
Supplement: Supplementary file 1 [file ijms-26-11052-s001.zip › ijms-3839292-supplementary.pdf]

SUPPLEMENTAL

Table S1. RIsk of Bias Assessment for Included Studies (Adapted from SYRCLE RoB Tool)

No	**Study (Author, Year)**	**Randomization**	**Blinding**	**EV Dose Quantification**	**Endpoint Validation**	**0verall Risk of Bias**
1.	Røsand et al. (2024) [39]	Unclear	Partial	No	Yes	Moderate
2.	keda et al. (2021) [16]	Yes	Partial (Blinding not fully reported)	Yes	Yes	Low
3.	Chen et al. (2025) [40]	Yes	Unclear	Yes	Yes	Moderate
4.	Zheng et al. (2024) [41]	Yes	No	Yes	Yes	Moderate
5.	Li et al. (2023) [42]	Yes	Yes	Yes	Yes	Low
6.	Yang et al. (2023) [43]	Yes	Yes	Yes	Yes	Low
7.	Wen et al. (2020) [44]	No	No	Yes	Yes	Moderate
8.	Liu et al. (2017) [45]	Unclear	No	Yes	Partial	Moderate
9.	Liu et al. (2024) [46]	Yes	Unclear	Yes	Yes	Moderate
10.	Liu X et al. (2024) [47]	Yes	Unclear	Yes	Yes	Moderate
11.	O'Brien et al. (2021) [48]	Yes	Yes	Yes	Yes	Low
12.	Liu, Dissanayaka, and Yiu (2025) [49]	N/A (Review)	N/A	N/A	N/A	High
13.	Kumar, Mehta, and Bissler (2023) [50]	N/A (Modeling paper)	N/A	N/A	N/A	High
14.	Heyn et al. (2023) [51]	N/A (Review)	N/A	N/A	N/A	High
15.	Chen and Liu (2023) [22]	N/A (Review)	N/A	N/A	N/A	High (Conceptual)
16.	Femminò, Bonelli, and Brizzi (2022) [53]	N/A (Review)	N/A	N/A	N/A	High
17	Chen et al. (2021) [54]	N/A (Review)	N/A	N/A	N/A	High (Conceptual)
18.	Ibáñez and Villena-Gutierrez (2021) [55]	Unclear	No	Yes	Yes	Moderate

**Low risk = Randomization + Blinding + EV dose + Validated outcomes clearly reported. Moderate risk = At least one of the four SYRCLE domains unclear or partially addressed. High risk = Conceptual / review/modeling papers or lacking experimental validation.*
